# Increased Prevalence of Unstable HLA-C Variants in HIV-1 Rapid-Progressor Patients

**DOI:** 10.3390/ijms232314852

**Published:** 2022-11-27

**Authors:** Chiara Stefani, Antonella Sangalli, Elena Locatelli, Tania Federico, Giovanni Malerba, Maria Grazia Romanelli, Gustavo Adolfo Argañaraz, Bosco Christiano Maciel Da Silva, Alberto Jose Duarte Da Silva, Jorge Casseb, Enrique Roberto Argañaraz, Alessandra Ruggiero, Donato Zipeto

**Affiliations:** 1 Section of Biology and Genetics, Department of Neurosciences, Biomedicine and Movement Sciences, University of Verona, 37134 Verona, Italy; 2 Lab of Molecular NeuroVirology, Faculty of Health Science, University of Brasília, Brasilia 70910-900, Brazil; 3 Medical Investigation Laboratory Unit 56 (LIM/56), Faculdade de Medicina FMUSP, University of São Paulo, São Paulo 05403-000, Brazil; 4 Faculty of Medicine, Institute of Tropical Medicine, University of São Paulo, São Paulo 05403-000, Brazil

**Keywords:** AIDS progression, HLA-C stability, HIV-1 infection control, ASPCR, MHC class I

## Abstract

HIV-1 infection in the absence of treatment results in progression toward AIDS. Host genetic factors play a role in HIV-1 pathogenesis, but complete knowledge is not yet available. Since less-expressed HLA-C variants are associated with poor HIV-1 control and unstable HLA-C variants are associated with higher HIV-1 infectivity, we investigated whether there was a correlation between the different stages of HIV-1 progression and the presence of specific HLA-C allotypes. HLA-C genotyping was performed using allele-specific PCR by analyzing a treatment-naïve cohort of 96 HIV-1-infected patients from multicentric cohorts in the USA, Canada, and Brazil. HIV-1-positive subjects were classified according to their different disease progression status as progressors (Ps, *n* = 48), long-term non-progressors (LTNPs, *n* = 37), and elite controllers (ECs, *n* = 11). HLA-C variants were classified as stable or unstable according to their binding stability to β2-microglobulin/peptide complex. Our results showed a significant correlation between rapid progression to AIDS and the presence of two or one unstable HLA-C variants (*p*-value: 0.0078, *p*-value: 0.0143, respectively). These findings strongly suggest a link between unstable HLA-C variants both at genotype and at allele levels and rapid progression to AIDS. This work provides further insights into the impact of host genetic factors on AIDS progression.

## 1. Introduction

Human immunodeficiency virus type 1 (HIV-1) is a lentivirus, a member of the *Retroviridiae* family, and it is the causative agent of the acquired immunodeficiency syndrome (AIDS) [1] in the absence of anti-retroviral treatment (ART). CD4+ T lymphocytes are the primary target of HIV-1, which infects cells through CD4 and CCR5/CXCR4 receptors, which leads to a depletion of CD4+ T lymphocytes and to an overall impairment of the immune system [2]. HIV-1 has spread because of a spillover event at the beginning of the 20th century and then became a global pandemic approximately in the 1980s. In 2020, global statistics on HIV-1 showed that there were 37.7 million people living with HIV-1, with 1.5 million people newly infected and 680,000 people dying from AIDS-related diseases [3,4]. To date, there is still no cure for HIV-1, but since the introduction of ART, the mortality rate associated with the development of AIDS and its comorbidities has significantly decreased. The speed of progression toward AIDS is highly variable among infected patients and depends on host genetic factors and HIV-1 variability [5,6]. Therefore, patients can be classified based on the HIV-1 progression rate into progressors (Ps), long-term non-progressors (LTNPs), and elite controllers (ECs). ECs and LTNPs are characterized by delayed AIDS progression, whereas Ps have a more rapid progression toward AIDS because of poor HIV-1 infection control [7,8,9].

Recently, attention has been turned to the study of human leukocyte antigen C (HLA-C) genetic variants, which are involved in HIV-1 infection control. The HLA-C gene (as well as HLA-A and -B) encodes for the α chain of the major histocompatibility complex class I (MHC-I). This trimeric complex consists of an HLA α-chain, β2-microglobulin (β2m), and an antigenic peptide; it is an essential component of the immune response because it presents intracellular peptides to CD8+ T lymphocytes. HLA genes are located on chromosome 6 and are among the most polymorphic genes in the human genome [10]. Besides HLA-C’s role in the antigen presentation process, it can bind killer-cell inhibitory receptors (KIRs) on NK cells, thus mediating their activation/inhibition [11,12].

Several studies on HIV-1 positive or AIDS patients have underlined how some HLA -A or -B alleles have a protective or deleterious role in HIV-1 infection control and progression to AIDS [13]. Moreover, having an HLA homozygous genotype is linked to worse control of HIV-1 infection and a faster AIDS progression [14]. Different HLA variants possess different peptide-binding affinities; thus, a heterozygous genotype allows a wider peptide range that could be presented to CD8+ T lymphocytes [15]. HLA-A and -B are more variable and more expressed than HLA-C, which instead binds less efficiently β2m, leading to a reduced HLA-C surface expression [16]. HIV-1 down-modulates HLA-A and -B through the HIV-1 protein Nef, while HLA-C is down-modulated by the action of the viral protein Vpu [17]. Nef and Vpu are members of HIV-1 accessory proteins: Nef is involved in CD4 and MHC-I downregulation, while Vpu inhibits NF-kB activation and has a role in peroxisomes downregulation [18,19,20]. HLA-C expression levels are also post-transcriptionally regulated. In particular, the presence of a SNP (rs67384697 G ins/del) in the 3′ untranslated region controls miR-148 binding efficacy (miR-148 binds to the +G allele), affecting HLA-C expression [21,22]. Some HLA-C alleles possess an intact binding site for miR-148, leading to a decreased expression, whereas others have a deletion that prevents miR-148 binding and have higher expression levels [23]. Moreover, HLA-C alleles possess different binding stability to the complex β2m/peptide [24]. Unstable HLA-C variants (HLA-C *01, *03, *04, *07, *14, *17, and *18) have a higher β2m dissociation rate, leading to the development of HLA-C “free chains” on the cell surface, which can be exploited by the HIV-1 Env protein to enhance viral infectivity. On the other hand, stable HLA-C variants (HLA-C *02, *05, *06, *08, *12, *15, and *16) are strongly bound to β2m, thus reducing “free chains” formation [25,26].

The aim of this work was to examine HLA-C’s impact on HIV-1 infection advancement and progression toward AIDS. We could benefit from having a unique cohort of 96 HIV-1-positive treatment-naïve patients. This study investigated the correlation between unstable HLA-C variants and AIDS progression, giving new insights and broadening knowledge that could be useful for HIV-1 treatment approaches.

## 2. Results

### 2.1. Studied Patient’s Cohort

The 96 enrolled subjects represent a unique cohort, since all the recruited patients were naïve of treatment at the time of sample collection and they managed HIV-1 infection in the absence of ART therapy. Based on their immuno-virological characteristics, patients were divided into 48 Ps, 37 LTNPs, and 11 ECs. General information of the studied cohort is reported in Table 1. The study population did not show a statistically significant deviation from Hardy–Weinberg equilibrium (*p*-value = 0.13). When the test was performed on the three separate groups, only progressors showed a statistically significant deviation from Hardy–Weinberg equilibrium compared to the other two categories (Ps: *p*-value = 0.023; LTNPs: *p*-value = 0.82; ECs: *p*-value = 0.98). A statistically significant difference between LTNPs and ECs median age was identified (Mann–Whitney test, *p*-value = 0.0143), whereas statistically significant differences between the distribution of male and female and the ethnicity in the three categories were not observed (chi-square test). On the contrary, there was a statistically significant difference regarding the sample origin (i.e., Brazil, Canada, and the USA) (chi-square test, *p*-value = 0.0009) between the three analyzed groups. The distribution of stable and unstable alleles was not statistically different (*p*-value 0.3382) in the sample cohort (i.e., Brazil, Canada, and the USA) (Table 2).

### 2.2. Immuno-Virological Characteristics of the Studied Cohort

The immuno-virological features, which lead to the patient’s classification, are reported in Table 3.

**Figure 1:**
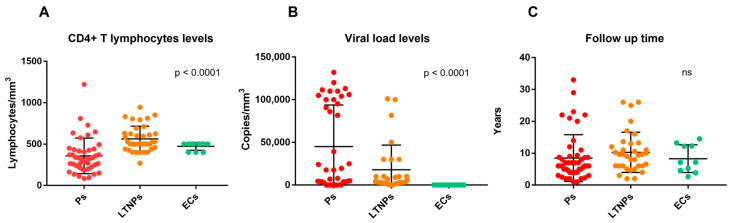
CD4+ T lymphocytes levels (**A**), viral load levels (**B**), and follow up time (**C**) in progressors (Ps), long-term non-progressors (LTNPs), and elite controllers (ECs). *p*-values computed through the Kruskal–Wallis test.

The analysis of CD4+ T lymphocytes and viral load levels using the Kruskal–Wallis test showed a statistically significant difference between the groups studied (*p* < 0.0001) for both parameters (Figure 1A,B). With regard to follow-up time, the Kruskal–Wallis test did not reveal a statistically significant difference (Figure 1C).

The immuno-virological data confirm that progressors have higher HIV-1 and lower CD4+ T lymphocytes levels (both parameters are distinctive traits of a worse HIV-1 control and a faster AIDS progression) as expected, whereas LTNPs and ECs have lower HIV-1 and higher CD4+ T lymphocytes levels, indicating a better HIV-1 infection control.

### 2.3. Unstable HLA-C Variants Correlate with HIV-1 Progression

We calculated the sample size and the statical power of the study in the case of allele association under the assumptions that unstable alleles increase the progression risk by 20% in heterozygous subjects and 40% in homozygous ones, and that the prevalence of patients with rapid progression is 85% and the frequency of unstable alleles in the general population (HIV-infected individuals) is 60%. Under these unknown but likely assumptions, the study showed to be powered enough (beta-value threshold = 0.8 and alpha-value threshold = 0.05) when the number of controls and cases is equal or greater than 43 subjects (i.e., 43 cases and 43 control individuals), indicating that the sample size is adequate for the main analysis reported in this study. Furthermore, we performed a logistic regression analysis to adjust for age and sex and found that these parameters are irrelevant for improving the risk prediction model (*p*-value = 0.68).

**Figure 2:**
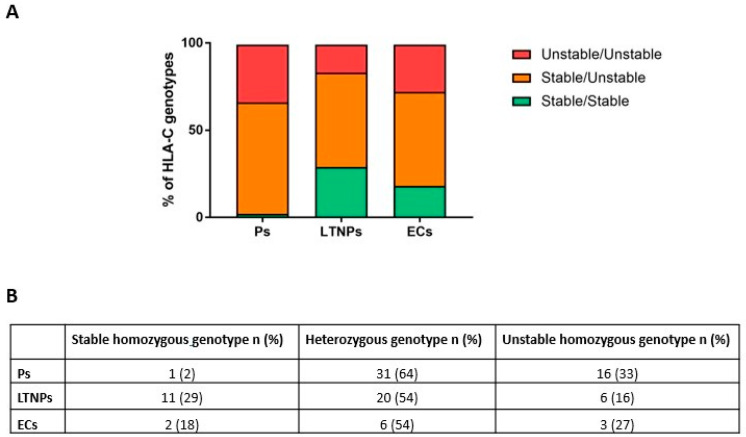
Graphical percentage distribution of HLA-C genotypes for each category of HIV-1-positive patients (**A**). Number and percentage of subjects for each category (**B**). *p*-value computed through chi-square test.

We performed the chi-square analysis both on genotypes (sorted as homozygous or heterozygous for stable/unstable HLA-C alleles) and on HLA-C alleles (as stable or unstable variants). The statistical analyses showed a significant association between genotype distributions (*p*-value 0.0078) (Figure 2).

**Figure 3:**
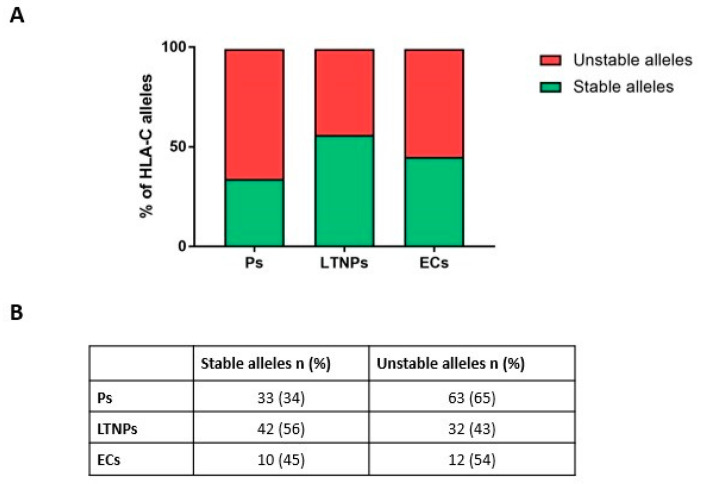
Graphical percentage distribution of stable and unstable HLA-C alleles frequency for each category of HIV-1-positive patients (**A**). Number and percentage of subjects for each category (**B**). *p*-value computed through chi-square test.

The analysis of the correlation between HLA-C alleles and HIV-1 progression showed a significant association (*p*-value 0.0143) in the whole cohort (Figure 3). Moreover, after stratifying by population origin, the same trend was observed in Canadian (*p*-value 0.0128) and USA (*p*-value 0.0171) patients, whereas a statistically significant association was not observed in Brazilian patients (*p*-value 0.6538).

## 3. Discussion

HLA-C has been widely studied for its pivotal role in the immune cellular response to viral infections. Previously published studies linked specific HLA-C variants to HPV-related cervical disease [27] to patients chronically infected by HCV [28] and by HBV [29]. The focus of this work was to determine whether there was an association between HLA-C variants’ stability and HIV-1 infection control. To this aim, a unique cohort of 96 HIV-1-infected treatment-naïve patients with different ethnic backgrounds were enrolled in three multicenter cohorts and classified as Ps, LTNPs, and ECs based on their immuno-virological parameters, which reflect their HIV-1 infection advancement. Progressors are characterized by lower CD4+ T lymphocytes, higher viral load levels, and a rapid progression to AIDS, whereas LTNPs and ECs possess higher CD4+ T lymphocytes and lower viral load levels, which lead to better control of HIV-1 infection. The statistical analysis of HLA-C patient’s genotypes classified as homozygous for stable/unstable alleles or heterozygous indicates that there is a strong association between unstable HLA-C genotypes and a worse control of HIV-1 infection (*p*-value 0.0078). Additionally, the analysis of HLA-C alleles (classified as stable or unstable variants) led to the identification of a statistically significant association between unstable HLA-C alleles and a more rapid disease advancement (*p*-value 0.0143). Furthermore, we observed that progressors are not in Hardy–Weinberg equilibrium (*p*-value = 0.023). This result underlines that unstable alleles may play a role in HIV-1 progression, since we observed an enrichment of unstable alleles in progressors, which represents the category that has a worse HIV-1 infection control. We observed that LTNPs and ECs possess 43% and 54% unstable alleles, respectively. It should be considered that HLA-C stability is not a binary parameter, but there is a continuous range of stability [25]. The determination of the exact stability of each individual HLA-C allele needs further investigation. In addition to HLA-C, there are multiple host and viral factors that may contribute to the control of HIV-1 infection. Previous studies published by our group reported that HIV-1 virions produced in the presence of unstable HLA-C alleles are more infectious than those produced in the presence of stable ones [25], and that a higher frequency of unstable HLA-C alleles was observed in subjects with HIV-1-associated neurocognitive disorders [30]. Another element that can influence HIV-1 infection control is HLA-C expression levels. It has been reported that HLA-C variants that possess higher expression levels were associated with better HIV-1 infection control [31]. HLA-C expression levels are a consequence of the presence/absence of the miR-148 binding site on the 3′ UTR of the HLA-C gene. HLA-C alleles that possess the intact miR-148 binding site are characterized by lower expression levels than those variants that do not possess the intact binding site [21]. In another published work, it has been reported that HLA-C expression levels correlate with a higher cytotoxic T lymphocyte response and with HIV-1 infection control [31]. Here we report that stable HLA-C alleles associate with slower progression toward AIDS. Since the most stable HLA-C alleles are generally among the more expressed ones (such as HLA-C *06:02, *12:03, and *08:02) and the most unstable HLA-C alleles are among the less expressed ones (such as HLA-C *03:02, *07:01/02, and *17), we hypothesize that both expression levels and stability may contribute to HIV-1 infectivity modulation [24,31]. However, there are some discrepancies between HLA-C stability and HLA-C expression levels: for example, HLA-C *04 is among the more expressed HLA-C alleles but is considered as an unstable variant. This divergence underlines the need of a more detailed analysis of the stability degree of every single HLA-C allele, which could lead to the improvement of the simple “binary” division between stable and unstable HLA-C alleles by considering the contribution of the stability and the expression levels of every single allele. Our results, reporting for the first time that unstable HLA-C variants are associated with faster AIDS progression, are consistent and support previously published data indicating that unstable/less-expressed HLA-C alleles are correlated to worse control of HIV-1 infection [25,31]. Among the limitations of this study is the reduced number of subjects analyzed, considering the highly mixed ethnic diversity of HIV-1 patients studied. Our data highlight the importance of host genetic factors in the progression of HIV-1 infection. We are aware of the difficulty in identifying a possible complex genotype that unequivocally characterizes the tendency to develop AIDS. However, if our data are confirmed by further analysis that considers a larger sample size of patients, this could represent an additional important piece to this complex puzzle. This genotype analysis could have an impact on the follow-up of HIV-1-infected patients, as the presence of unstable alleles correlates with faster progression. In light of this information, HIV-1-infected patients with an unstable homozygous HLA-C genotype could be monitored more frequently and/or start therapy earlier. Considering that unstable HLA-C variants are associated with worse control of HIV-1 infection, taking the HLA-C genotype into account could contribute to the development of new personalized therapeutic approaches for HIV-1.

## 4. Materials and Methods

### 4.1. Study Population

Ninety-six HIV-1 patients from three multicentric cohorts (the USA, Canada, and Brazil) were enrolled in this study and classified according to clinical disease progression as EC (50–1000 copies HIV genome/mm^3^, and ≥400 CD4+ T lymphocytes/mm^3^), LTNP (<10,000 copies HIV genome/mm^3^, and ≥400 CD4+ T lymphocytes/mm^3^), and P (>10,000 copies HIV genome/mm^3^, and ≤200 CD4+ T lymphocytes/mm^3^). The majority of subjects analyzed were infected with HIV-1 clade B. CD4+ T-cell counts, and HIV viral-load values were determined periodically during the patient’s follow-up time (i.e., the time between the detection of the infection and the end of the follow-up). In the case of ECs and LTNPs, the measurements were always >400 T lymphocytes/mm^3^. In the case of progressors, CD4+ T-cell counts decreased during follow-up. When progressor patients showed a decrease in the lymphocyte count to less than ≤200 CD4+ T-cell count during follow-up, treatment was started and sample collection was stopped.

All subjects gave informed consent, and the research protocols were approved by the corresponding institutional review boards’ research ethics committees: Núcleo de Medicina Tropical (# 275.456/2013), from the Federal University of Pará Brazil; the Ethics Committee of the Health Department of the Federal District (#066/07) from hospitals of the public network of the Federal District, Brazil; the Ethics Committee for Analysis of Research Projects (CAPPesq) of Hospital das Clínicas HCFMUSP (#CAPPesq #0306/10, online registration #5867), Faculdade de Medicina da Universidade de Sao Paulo, Brazil. The 96 enrolled patients were subdivided into 48 progressors (Ps), 37 long-term non-progressors (LTNPs), and 11 elite controllers (ECs). In addition, all 96 subjects enrolled in this study were treatment naïve at the time of sampling, which makes this cohort unique.

### 4.2. DNA Extraction and HLA-C Genotyping

Genomic DNA from peripheral blood mononuclear cells was obtained using the phenol–chloroform extraction method. Briefly, the procedure was performed in the following steps: (1) red blood cell lysis with solution A (ammonium chloride 1.0 M, ethylenediaminetetraacetic acid (EDTA) 1.0 M) and solution B (ammonium bicarbonate 1.0 M); (2) lysis of leukocytes (Tris-HCl 100 mM, EDTA 20 mM, NaCl 200 mM, 0.5% sodium dodecyl sulfate (SDS)); (3) protein precipitation (ammonium acetate 7.5 M); and (4) DNA hydration (distilled water free of DNase and RNase).

HLA-C genotyping was performed through allele-specific PCR (ASPCR). ASPCR reactions were carried out using Wonder Taq (Euroclone) and run on a LifeTouch thermal cycler (Bioer) using previously published primer pairs [32,33]. To detect false negative results, an internal control gene was co-amplified. Collagen type V alpha 1 chain (COL5A1) and methylthioadenosine phosphorylase (MTAP) were selected and amplified, employing previously published primer pairs [34,35]. PCR conditions were set up for each HLA-C-tested allele. When the HLA-C genotype could not be uniquely determined by ASPCR, the HLA-C region between exon 2 and 3 was amplified using Wonder Taq (Euroclone) run on a LifeTouch thermal cycler (Bioer), followed by Sanger sequencing. The primer pairs used for amplification (5CIn1-61 and 3BCIn3-12) and Sanger sequencing (CEx2F) were previously described [36]. Sequencing was performed by BMR Genomics (https://www.bmr-genomics.it/) and the obtained sequences were analyzed using a bioinformatic approach: HLA-C allele combinations underlining the undetermined genotype were then detected by a phasing procedure, including the 4125 genomic allele reference sequences available in the IMGT database (“https://www.ebi.ac.uk/ipd/imgt/hla/”; IPD-IMGT/HLA database, last download date: 6 December 2021).

### 4.3. Statistical Analysis

The nonparametric Mann–Whitney T-test, Kruskal–Wallis test, chi-square test, and the descriptive statistics were performed using GraphPad Prism version 7.03 for Windows, GraphPad Software, San Diego, CA, USA, www.graphpad.com. The chi-square statistical analysis of patients’ genotypes was performed using a 3 × 3 contingency table, whereas the analysis on HLA-C alleles was conducted using a 3 × 2 contingency table. The obtained *p*-values were considered significative when the computed values were lower than 0.05. A logistic regression model was run taking age and sex as covariates; models with and without covariates were compared using ANOVA test.

## Figures and Tables

**Table 1:** General characteristics of the studied patient cohort.

		Ps (*n* = 48) *	LTNPs (*n* = 37) *	ECs (*n* = 11) *	*p*-Value
Age, median (IQR), years		40.5 (30–52.5)	43 (33.5–52.5)	33 (27–41)	Ps vs. LTNPs *p* = 0.438 Ps vs. ECs *p* = 0.112 ECs vs. LTNPs *p* = 0.0143
Male, *n* (%)		37 (77)	27 (72.9)	7 (63.6)	*p* = 0.6471
Female, *n* (%)		11 (23)	10 (27.1)	4 (36.4)	
Ethnicity, *n* (%)	Caucasian	44 (91.6)	31 (83.8)	7 (63.6)	*p* = 0.0558
	Black	4 (8.4)	6 (16.2)	4 (36.4)	
Origin, *n* (%)	Brazil	28 (58.4)	19 (51.4)	-	*p* = 0.0009
	Canada	14 (37.8)	14 (37.8)	11 (100)	
	USA	6 (12.5)	4 (10.8)	-	

* Ps indicated Progressors; LTNPs indicates Long-Term Non Progressors; ECs indicates Elite Controllers.

**Table 2:** Distribution of stable and unstable alleles in the studied cohort.

	Stable Alleles	Unstable Alleles
Brazil	37	57
Canada	37	41
USA	11	9

**Table 3:** Immuno-virological characteristics of the studied cohort.

	Ps (*n* = 44) *	LTNPs (*n* = 37) *	ECs (*n* = 11) *	***p*-Value**
CD4, median (IQR), lymphocytes/mm^3^	324 (219.5–431)	500 (451–627)	500 (400–500)	Ps vs. LTNPs *p* < 0.0001 Ps vs. ECs *p* = 0.0017 ECs vs. LTNPs *p* = 0.0528
	Ps (*n* = 39) *	LTNPs (*n* = 29) *	ECs (*n* = 11) *	
Viral load, median (IQR), copies/mm^3^	17,698 (3688–100,000)	4119 (2691–20,000)	50 (50–50)	Ps vs. LTNPs *p* = 0.0773 Ps vs. ECs *p* < 0.0001 ECs vs. LTNPs *p* < 0.0001
	Ps (*n* = 48) *	LTNPs (*n* = 35) *	ECs (*n* = 10) *	
Follow up, median (IQR), years	6 (4–8.9)	9.6 (6–13)	7.14 (4.4–12.5)	Ps vs. LTNPs *p* = 0.0235 Ps vs. ECs *p* = 0.505 ECs vs. LTNPs *p* = 0.513

* Ps indicates progressors; LTNPs indicates long-term non-progressors; ECs indicates elite controllers.

## Data Availability

Data will be made available upon request.
